# *Chlamydia psittaci* Plasmid-Encoded CPSIT_P7 Elicits Inflammatory Response in Human Monocytes via TLR4/Mal/MyD88/NF-κB Signaling Pathway

**DOI:** 10.3389/fmicb.2020.578009

**Published:** 2020-12-03

**Authors:** Qian Chen, Yumeng Li, Xiaoliang Yan, Zhenjie Sun, Chuan Wang, Shuangquan Liu, Jian Xiao, Chunxue Lu, Yimou Wu

**Affiliations:** ^1^Institution of Pathogenic Biology, Hengyang Medical College, University of South China, Hunan Provincial Key Laboratory for Special Pathogens Prevention and Control, Hunan Province Cooperative Innovation Center for Molecular Target New Drug Study, University of South China, Hengyang, China; ^2^Institute of Clinical Research, The First Affiliated Hospital of University of South China, Hengyang, China; ^3^Department of Clinical Laboratory, The First Affiliated Hospital of University of South China, Hengyang, China

**Keywords:** *Chlamydia psittaci*, CPSIT_P7, human monocytes, TLR4, inflammation

## Abstract

The chlamydial plasmid, an essential virulence factor, encodes plasmid proteins that play important roles in chlamydial infection and the corresponding immune response. However, the virulence factors and the molecular mechanisms of *Chlamydia psittaci* are not well understood. In the present study, we investigated the roles and mechanisms of the plasmid-encoded protein CPSIT_P7 of *C. psittaci* in regulating the inflammatory response in THP-1 cells (human monocytic leukemia cell line). Based on cytokine arrays, CPSIT_P7 induces the expression of interleukin-6 (IL-6), interleukin-8 (IL-8), and monocyte chemoattractant protein-1 (MCP-1) in THP-1 cells. Moreover, the expression levels of IL-6, IL-8, and MCP-1 stimulated by CPSIT_P7 declined after silencing of the Toll-like receptor 4 (TLR4) gene using small interfering RNA and transfection of a dominant negative plasmid encoding TLR4 (pZERO-hTLR4). We further demonstrated that transfection with the dominant negative plasmid encoding MyD88 (pDeNy-hMyD88) and the dominant negative plasmid encoding Mal (pDeNy-hMal) could also abrogate the expression of the corresponding proteins. Western blot and immunofluorescence assay results showed that CPSIT_P7 could activate nuclear factor κB (NF-κB) signaling pathways in THP-1 cells. Altogether, our results indicate that the CPSIT_P7 induces the TLR4/Mal/MyD88/NF-κB signaling axis and therefore contributes to the inflammatory cytokine response.

## Introduction

*Chlamydia psittaci* is an intracellular pathogen of zoonotic pathogens responsible for atypical pneumonia. It can also lead to psittacosis or ornithosis in birds and economic losses in poultry farming (Voigt et al., 2012; Radomski et al., 2016; Wang et al., 2019). The preferred hosts of *C. psittaci* are wild birds, psittacine birds, and poultry (Bovijn et al., 2013), but dogs, cats, sheep, swine, cattle, and horses can also be infected (Pantchev et al., 2010). Humans often develop serious respiratory diseases such as pneumonia, after infection from the above animals (Jordan et al., 2003; Longbottom and Coulter, 2003). The microbes can spread to other tissues and present severe systemic infection that ranges from inapparent to severe (Liang et al., 2016). After successful infection, the disease mainly presents as headache, chills, malaise, and myalgia (Harkinezhad et al., 2009), or even death in untreated patients. Although *C. psittaci* is a threatening zoonotic pathogen due to its prevalence and potential threat, the pathogenesis of *C. psittaci* is still unclear.

*Chlamydia psittaci* 6BC possesses a plasmid 7553 bp in size that is predicted to harbor eight coding sequences, designated Pgp1–8 (Pawlikowska-Warych et al., 2015). Although the function of the plasmid has not been definitively proven, evidence has shown that plasmid deficiency fails to induce chlamydial immunopathology, manifesting the crucial role of the plasmid in chlamydial pathogenesis (O’Connell et al., 2011; Frazer et al., 2012; Zhou et al., 2013). The *C. psittaci* plasmid-encoded protein CPSIT_P7 (Pgp3) is homologous to the plasmid Pgp3 of *Chlamydia trachomatis* and *Chlamydia muridarum*, with a sequence identity of 70% and 71%, respectively. The conservative sequences of *C. trachomatis* strains differ compared to plasmids of *C. psittaci* by substitution of two nucleotide sequences. One of the major frames is ORF5, which encodes the 28-kDa protein Pgp3, for a marker of chlamydial infections (Harkinezhad et al., 2009). In murine models, plasmid-mediated virulence is known to trigger Toll-like receptors (TLRs) to boost proinflammatory cytokines (O’Connell et al., 2011). It has been reported that the *C. trachomatis* Pgp3 protein contributes to the inflammatory processes associated with chlamydial infections (Abdelrahman and elland, 2005). It is worth noting that *C. muridarum* Pgp3 is an effective virulence factor that can induce hydrosalpinx in mice and lacking Pgp3 may mediate the attenuation of *C. muridarum* pathogenicity (Liu et al., 2014). Although pathogenesis of Pgp3 in *C. trachomatis* and *C. muridarum* has been done in many researches, the precise biological function of CPSIT_P7 in *C. psittaci* is still largely unknown.

TLRs play a crucial role in activating innate immune cells including monocytes, macrophages, and dendritic cells. They can recognize related structures in pathogens, called pathogen-associated molecular patterns (PAMPs), and then initiate signaling cascades (Xie et al., 2017). To date, 10 types of TLRs in humans have been identified (TLR1-10), and all 10 TLRs are expressed in human monocytes. Except for TLR3, all TLRs use MyD88 for activation of NF-κB to produce inflammatory reactions (Pandey et al., 2014; Jafari et al., 2017; Li et al., 2018; Rodriguez-Valentin et al., 2018). TLR2, TLR4, and TLR6 signaling depends on the Toll-interleukin1 receptor (TIR) domain including adaptor protein (TIRAP), also referred to as Mal (MyD88 adapter-like), which mediates the interaction between MyD88 and activated TLRs (Pandey et al., 2014). The excitation of multiple signaling pathways by TLRs can activate transcription factors, such as NF-κB, and transcription factors can prevent pathogen infection in the host by regulating the expression of cytokines and chemokines (Aderem and Ulevitch, 2000).

The main proinflammatory cytokines, IL-6, IL-8, and MCP-1(CCL2) are the key chemokines produced by different types of cells, such as monocytes, macrophages, and endothelial and epithelial cells (Deshmane et al., 2009; Klapproth and Sasaki, 2010; Yang et al., 2018; Kanmani et al., 2019). Proinflammatory cytokines play an important role in host immunity against pathogen invasion by recruiting inflammatory cells and triggering immune responses. Moreover, IL-6, IL-8, and MCP-1 have been identified as the key factors for induction and are involved in various inflammatory diseases, which are also associated with the severity of inflammatory diseases (Deshmane et al., 2009; Klapproth and Sasaki, 2010; Kanmani et al., 2019). Previous studies revealed that *C. psittaci* stimulated various inflammatory factors in monocytes (He et al., 2015). In addition, infection with *C. psittaci* could also upregulate inflammatory mediators such as IL-6 and IL-8 in different cells containing the cervical epithelioid carcinoma HeLa cell line and the cervical squamous carcinoma SiHa cell line (He et al., 2015). Moreover, *in vitro* studies have shown that MCP-1 can be produced in the human endothelial cells after chlamydial infection, which is a leading contributor of atherosclerotic lesions characterized by monocyte infiltration (Molestina et al., 2000). In a recent study, MCP-1 deficiency was identified as less prone to lead to experimental atherosclerosis in mice (Gu et al., 1998; Gosling et al., 1999). However, the available studies have also underscored the current lack of a complete understanding of the relationship between the secretion of inflammatory cytokines and *C. psittaci* challenge, which is crucial for the pathogenesis of chlamydia.

Given the significance of plasmid-encoded proteins in the innate immune response and the inflammatory response during chlamydial infections, we mainly focused on investigating the role and molecular mechanism of CPSIT_P7 in the inflammatory response in human monocytic cells.

## Results

### Cytokine Expression in Conditioned Medium From THP-1 Cells Co-cultured With CPSIT_P7

**FIGURE 1:**
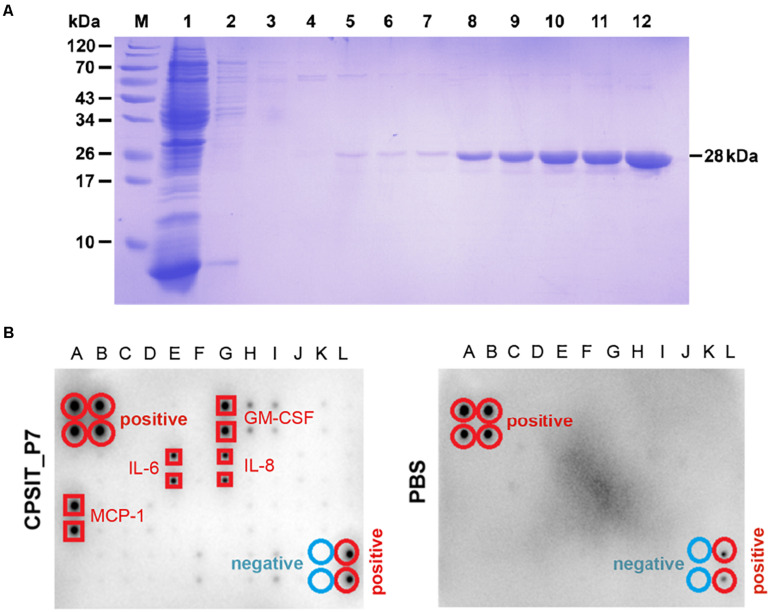
Purification of CPSIT_P7 protein and cytokine level analysis. **(A)** SDS-PAGE analysis of recombinant protein CPSIT_P7 purification. Lane M, pre-stained protein marker; Lane 1: flow through; Lanes 2–7: 10, 20, 30, 50, and 50 mM imidazole washing buffer, respectively. Lanes 8–12: 100, 150, 150, 250, and 250 mM imidazole eluting buffer, respectively. The recombinant CPSIT_P7 protein with a predicted size of 28 kDa. **(B)** THP-1 cells were stimulated with PBS (control) or CPSIT_P7 (10.0 μg/ml, 12 h). Culture supernatants were collected, pooled (*n* = 3/group), and screened for inflammatory cytokine, and then detected by a human cytokine antibody array. Signals were quantified and the cytokines are listed. IL-6 and IL-8: interleukin 6 and 8; MCP-1: monocyte chemotactic protein 1.

We initially tested the activity of CPSIT_P7 and stimulated the production of various inflammatory cytokines in THP-1 cells. The purity of CPSIT_P7 protein was detected by SDS-PAGE, and analysis revealed it to have a purity of >95% (Figure 1A). To define secreted cytokines, we used human cytokine antibody arrays to detect cytokine expression in conditioned media prepared from CPSIT_P7-stimulated THP-1 cells. The signal intensities for CPSIT_P7 protein were normalized to the samples treated with PBS (Figure 1B). Among the 42 cytokines tested, the four cytokines mainly secreted by THP-1 cells were granulocyte-monocyte colony-stimulating factor (GM-CSF), IL-6, IL-8, and MCP-1 (Figure 1B), which indicated that the increased levels of inflammatory molecules may contribute to inflammation *in vitro*.

### CPSIT_P7 Induces IL-6, IL-8, and MCP-1 Expression in a Concentration- and Time-Dependent Manner

**FIGURE 2:**
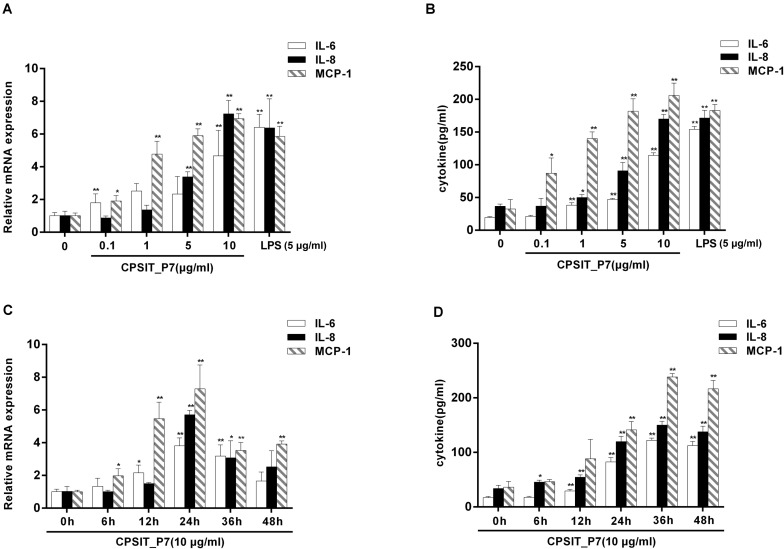
CPSIT_P7 induces IL-6, IL-8, and MCP-1 expression in a concentration- and time-dependent manner. THP-1 cells were stimulated with CPSIT_P7 (0, 0.1, 1, 5, or 10 μg/ml) for 24 h or treated with 10 μg/ml CPSIT_P7 for different time points (0, 6, 12, 24, 36, or 48 h). The gene and protein expression levels of IL-6, IL-8, and MCP-1 were analyzed by RT-qPCR **(A,B)** and ELISA **(C,D)**, respectively. Data are the means ± SD of three independent experiments. *P < 0.05, **P < 0.01 vs. the corresponding control.

To define the ability of CPSIT_P7 to stimulate IL-6, IL-8, and MCP-1 expression, concentration and time course experiments were performed. THP-1 cells were stimulated with different concentrations of CPSIT_P7 for 24 h or were treated with 10.0 μg/ml CPSIT_P7 for different time periods. As shown in Figure 2A, the expression levels of the IL-6, IL-8, and MCP-1 genes were obviously upregulation when cells were treated with 10 μg/ml CPSIT_P7 in a concentration-dependent manner. Similar to the previous results, the protein expression levels of IL-6, IL-8, and MCP-1 were markedly increased (Figure 2B). Figure 2C shows that CPSIT_P7 stimulation began to increase IL-6, IL-8, and MCP-1 mRNA expression levels at 6 h and continuously increased them up to 24 h. A similar trend was found with the protein expression levels of the inflammatory cytokines IL-6, IL-8, and MCP-1 released from THP-1 cells. At 36 h, the levels of the three target proteins reached peak values and then gradually decreased (Figure 2D). These situations were used for subsequent experiments.

**FIGURE 3:**
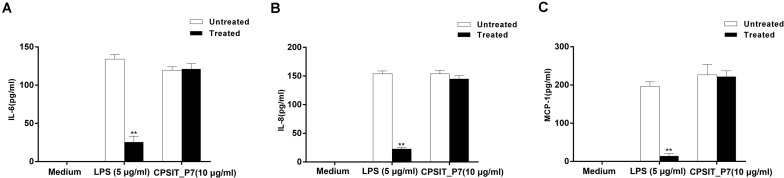
Effects of LPS on CPSIT_P7 induced IL-6, IL-8, and MCP-1 expression in THP-1 cells. After treatment with the Endotoxin Removal Kit to remove *E. coli* LPS, the levels of IL-6 **(A)**, IL-8 **(B)**, and MCP-1 **(C)** induced by CPSIT_P7 in THP-1 cells were detected by ELISA. The data shown are representative of three independent experiments. Data are means ± SD. **P < 0.01 vs. the control untreated with the Endotoxin Removal Kit.

In addition, the ability of Endotoxin Removal Kit-treated LPS to stimulate cytokine production in monocytes was significantly decreased compared with that of untreated LPS-stimulated monocytes, but untreated and treated CPSIT_P7 did not significantly stimulate the production of IL-6, IL-8, and MCP-1 (Figure 3), indicating that *E. coli* LPS contamination does not take part in the expression of IL-6, IL-8, and MCP-1 mediated by CPSIT_P7.

### CPSIT_P7 Induces IL-6, IL-8, and MCP-1 Production Through TLR4 but Not TLR2 and TLR6

**FIGURE 4:**
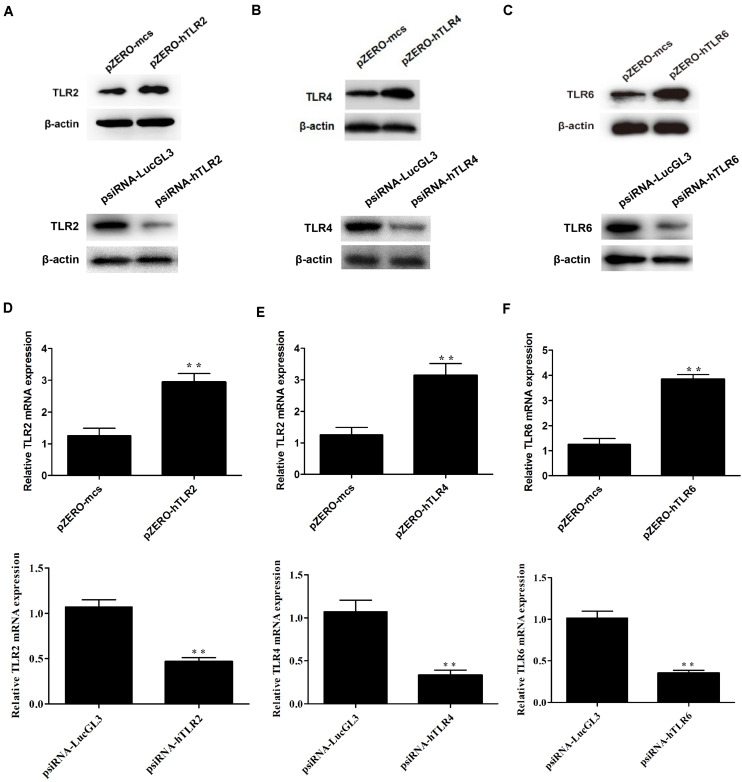
The expression levels of TLR2, TLR4, and TLR6 were detected after transfection with the dominant negative plasmids or psiRNA-hTLR plasmids. **(A–C)** The expression levels of TLR2, TLR4, and TLR6 protein were detected by Western blotting after transfection with the dominant negative plasmids (pZERO-hTLRs) and psiRNA-hTLRs. **(D–F)** The expression levels of TLR2, TLR4, and TLR6 mRNA were detected by RT-qPCR after transfected with the dominant negative plasmids (pZERO-hTLRs) and psiRNA-hTLRs. The data shown are representative of three independent experiments. Data are means ± SD. ^∗∗^*P* < 0.01 vs. psiRNA-LucGL3 control group.

To determine whether CPSIT_P7-induced THP-1 activation is TLR-dependent, the dominant negative plasmids TLR2 (pZERO-hTLR2), TLR4 (pZERO-hTLR4), TLR6 (pZERO-hTLR6), engineered with TIR domain-deleted TLR genes, were used to suppress the function of TLRs. In addition, the THP-1 cells also transfected with psiRNA-hTLRs plasmids expressing specific siRNA to suppress TLR expression. The result showed that the levels of TLR2, TLR4, and TLR6 proteins and mRNAs were upregulated in THP-1 cells after being transfected with pZERO-hTLRs (Figure 4). Compared with control psiRNA-LucGL3, the expression of TLR2, TLR4, and TLR6 proteins and mRNAs was significantly reduced in THP-1 cells after being transfected with psiRNA-hTLRs plasmids (Figure 4).

**FIGURE 5:**
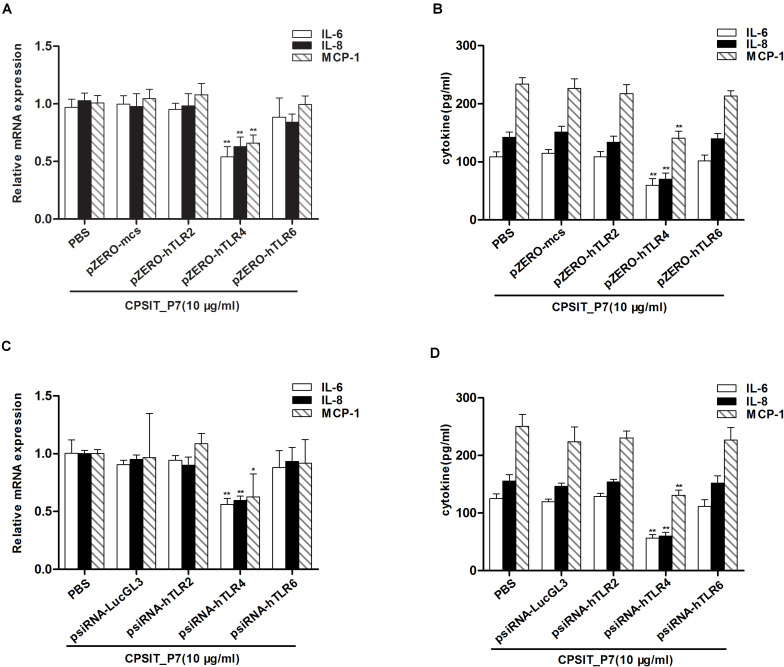
TLR4 is essential for CPSIT_P7-induced IL-6, IL-8, and MCP-1 expression. **(A,B)** THP-1 cells were transfected with pZERO-hTLR2, pZERO-hTLR4, and pZERO-hTLR6 for 48 h (pZERO-mcs was used as a control), and then stimulated with CPSIT_P7 (10.0 μg/ml, 24 h) to detect the expression levels of IL-6, IL-8, and MCP-1 mRNA or protein by RT-qPCR and ELISA. **(C,D)** THP-1 cells were transfected with psiRNA-hTLR2, psiRNA-hTLR4, and psiRNA-hTLR6 for 28 h (psiRNA-LucGL3 was used as a control), and then stimulated with 10 μg/ml CPSIT_P7 for 24 h to measure the mRNA or protein expression levels of IL-6, IL-8, and MCP-1 by RT-qPCR and ELISA. Data are the means ± SD of three independent experiments. *P < 0.05, **P < 0.01 vs. pZERO-mcs or psiRNA-LucGL3 negative control groups.

After inhibiting the function of TLR, THP-1 cells were treated with CPSIT_P7 (10.0 μg/ml, 24 h). The results showed that silencing of TLR4 by dominant negative plasmids could significantly downregulate the secretion levels of IL-6, IL-8, and MCP-1 in cells. However, silencing of TLR2 and TLR6 did not show any significant differences in downregulating the secretions of IL-6, IL-8, and MCP-1 compared to the level with control pZERO-mcs transfection (Figures 5A,B). In addition, as with the silencing of TLR by dominant negative plasmids, THP-1 cells transfected with psiRNA-hTLR4 could significantly inhibit the expression levels of IL-6, IL-8, and MCP-1 response to CPSIT_P7 (Figures 5C,D). These data suggested that TLR4 is involved in the production of IL-6, IL-8, and MCP-1 induced by CPSIT_P7 THP-1 cells. In addition, co-immunoprecipitation results showed that CPSIT_P7 could indeed combine with TLR4, and the binding complex was significantly reduced after sealing with TLR4 neutralizing antibody (Supplementary Figure S1).

### Mal Is Involved in CPSIT_P7-Induced IL-6, IL-8, and MCP-1 Expression

**FIGURE 6:**
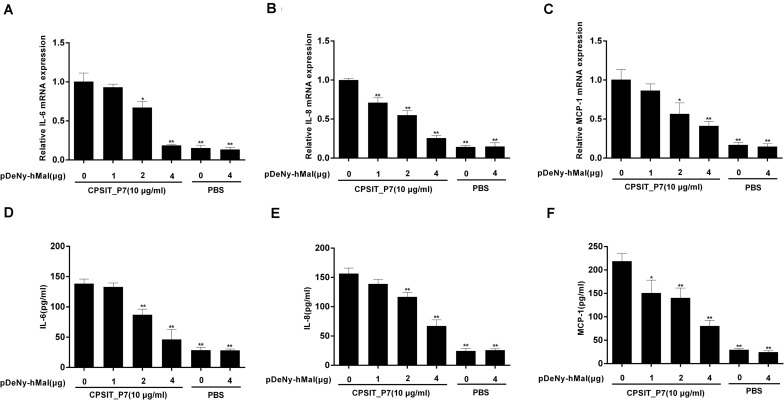
Effects of Mal on CPSIT_P7-induced IL-6, IL-8, and MCP-1 expression in THP-1 cells. THP-1 cells were transfected with 1, 2, or 4 μg of dominant negative plasmid encoding Mal (pDeNy-hMal) or empty vector (pDeNy-mcs) as a control for 48 h, and then stimulated with CPSIT_P7 (10.0 μg/ml, 24 h) to detect the expression levels of IL-6, IL-8, and MCP-1 mRNA by RT-qPCR **(A–C)**, or treated with CPSIT_P7 (10.0 μg/ml, 36 h) to detect the levels of IL-6, IL-8, and MCP-1 protein in cellular supernatants by ELISA **(D–F)**. The results shown are representative of three independent experiments. Data are means ± SD. *P < 0.05, **P < 0.01 vs. control group (10 μg/ml CPSIT_P7, 0 μg pDeNy-hMal).

Mal is an adapter protein that interacts with most TLRs and MyD88. THP-1 cells were transfected with the dominant negative plasmid pDeNy-hMal, which is a truncated form of Mal containing the C-terminal TIR domain but lacking the death domain. Compared with the cells transfected with the control plasmid pDeNy-mcs, the expression level of Mal was upregulated in cells transfected with pDeNy-hMal (data not shown). After inhibiting the function of Mal, CPSIT_P7 was applied to stimulate THP-1 cells (10.0 μg/ml, 24 h). The results showed that the IL-6, IL-8, and MCP-1 mRNA expression levels were significantly decreased when the cells were transfected with the pDeNy-hMal plasmid (Figures 6A–C). As described above, a similar trend was found in the protein expression levels of the inflammatory cytokines IL-6, IL-8, and MCP-1 released from THP-1 cells (Figures 6D–F).

### CPSIT_P7 Induces the Production of IL-6, IL-8, and MCP-1 via MyD88

**FIGURE 7:**
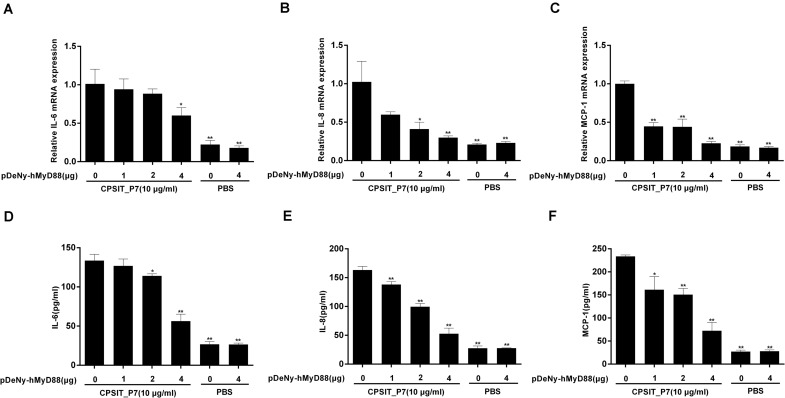
CPSIT_P7 induced the expression of IL-6, IL-8, and MCP-1 in THP-1 cells via MyD88. THP-1 cells were transfected with 1, 2, or 4 μg of dominant negative plasmid encoding MyD88 (pDeNy-hMyD88) or empty vector (pDeNy-mcs) as a control for 48 h and were treated with CPSIT_P7 (10.0 μg/ml, 24 h) to detect the levels of IL-6, IL-8, and MCP-1 mRNA by RT-qPCR **(A–C)**, or were treated with 10 μg/ml CPSIT_P7 for 36 h to detect the levels of IL-6, IL-8, and MCP-1 protein in the cellular supernatants by ELISA **(D–F)**. The results shown are representative of three independent experiments. Data are means ± SD. *P < 0.05, **P < 0.01 vs. control group (10 μg/ml CPSIT_P7, 0 μg pDeNy-hMyD88).

To investigate whether MyD88 is involved in CPSIT_P7-induced IL-6, IL-8, and MCP-1 production, we further transfected THP-1 cells with the dominant negative plasmid pDeNy-hMyD88 in THP-1 cells. The expression of MyD88 was significantly increased after transfection with pDeNy-hMyD88 in THP-1 cells (data not shown). After inhibiting the function of MyD88, the expression levels of IL-6, IL-8, and MCP-1 mRNA in THP-1 cells were markedly suppressed (Figures 7A–C). Similar trends were found with inflammatory cytokines such as IL-6, IL-8, and MCP-1 protein expression when the cells were treated with different concentrations of pDeNy-hMyD88 (Figures 7D–F). These results imply that the TLR pathway is involved in the induction of inflammatory response induced by CPSIT_P7 in THP-1 cells in a MyD88-dependent manner.

**FIGURE 8:**
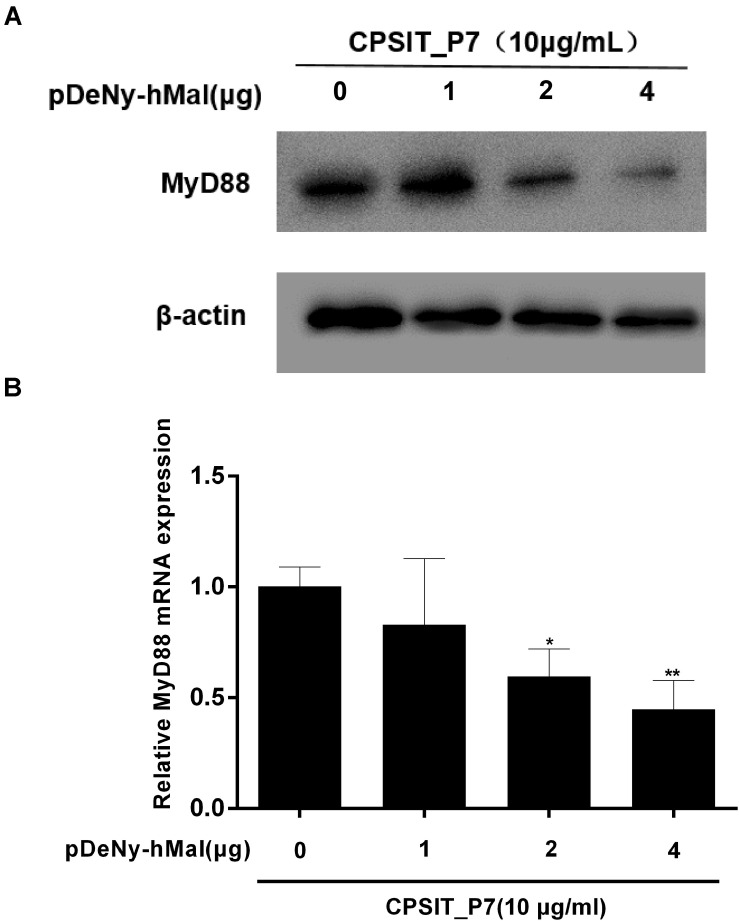
The expression of MyD88 protein and mRNA were downregulated in THP-1 cells after transfection with pDeNy-hMal. THP-1 cells were treated with different concentrations of the dominant negative plasmid encoding Mal (pDeNy-hMal) for 48 h and were then stimulated with CPSIT_P7 (10.0 μg/ml, 24 h) to measure the protein and mRNA expression levels of MyD88 by Western blotting **(A)** and RT-qPCR **(B)**. Data are the means ± SD of three independent experiments. *P < 0.05, **P < 0.01 vs. control group (0 μg pDeNy-hMal).

Furthermore, the expression of MyD88 protein and mRNA was significantly inhibited after transfection with pDeNy-hMal, compared to that in control untransfected cells stimulated with CPSIT_P7 (Figure 8). These results indicated that the Mal–MyD88 axis is involved in the CPSIT_P7-induced inflammatory response.

### CPSIT_P7 Promotes an Inflammatory Response via the Activation of NF-κB Activation

**FIGURE 9:**
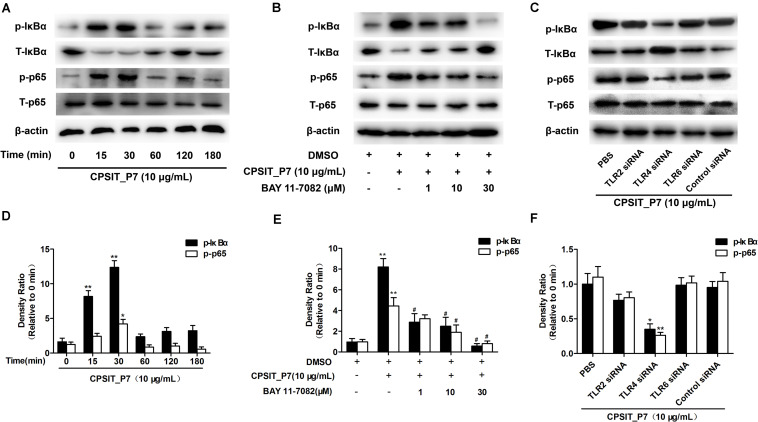
CPSIT_P7 induced the degradation of IκBα via the TLR4 pathway in THP-1 cells. **(A,D)** THP-1 cells were treated with CPSIT_P7 (10.0 μg/ml) for different time periods. The expression of IκBα was analyzed by Western blotting and quantified using ImageJ software. **(B,E)** THP-1 cells were pretreated with 1, 10, or 30 μM IκBα inhibitor BAY11-7082 for 30 min, and treated with 10 μg/ml CPSIT_P7 for 30 min. The expression of IκBα was analyzed by Western blotting and quantified by ImageJ software. **(C,F)** psiRNA-hTLR2, psiRNA-hTLR4, and psiRNA-hTLR6 were transfected into THP-1 cells, and then stimulated with CPSIT_P7 (10.0 μg/ml) for 30 min. The expression of IκBα was analyzed by Western blotting and quantified using ImageJ software. *P < 0.05, **P < 0.01 vs. control groups, ^#^*P* < 0.05 vs. CPSIT_P7 (10.0 μg/ml) + BAY 11-7082 (−) group.

It is well known that activation of NF-κB is a pivotal step in TLR-mediated responses and is essential to induce the innate immune response to pathogens, eventually resulting in the production of inflammatory cytokines. Therefore, we investigated the degradation level of IκBα in THP-1 cells after stimulation with CPSIT_P7. As shown in Figures 9A,D, the phosphorylation of IκBα reached a peak at 30 min and then decreased after treatment with CPSIT_P7. Furthermore, the phosphorylation of IκBα was inhibited when the cells were pretreated with the IκBα inhibitor BAY11-7082 (Figures 9B,E). The THP-1 cells were transfected with psiRNA-hTLR2, psiRNA-hTLR4, psiRNA-hTLR6, and stimulated with 10 μg/ml CPSIT_P7 to analyze the phosphorylation of IκBα. The results revealed that the phosphorylation of IκBα was significantly inhibited after cells were transfected with psiRNA-hTLR4 (Figures 9C,F). These results indicated that CPSIT_P7 could activate the NF-κB signaling pathway via TLR4 in THP-1 cells.

**FIGURE 10:**
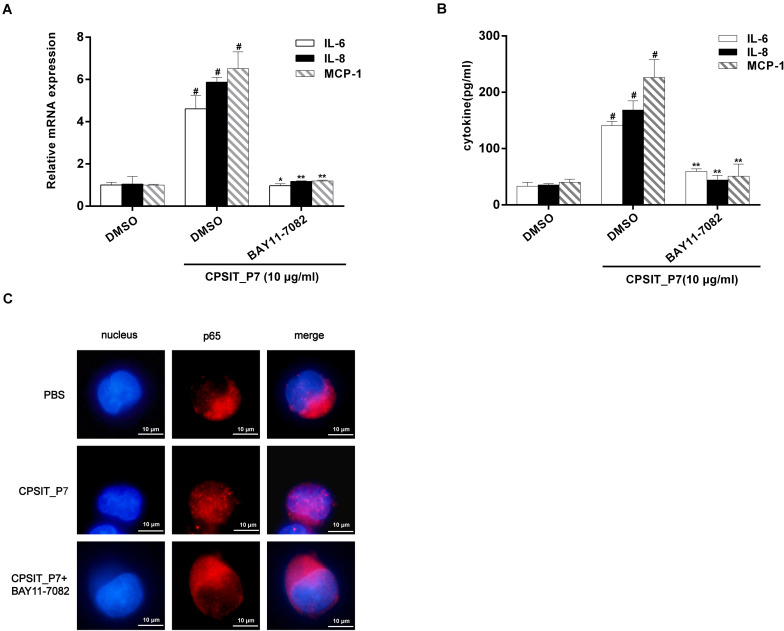
The NF-κB signaling pathway is essential for CPSIT_P7-induced IL-6, IL-8, and MCP-1 expression. THP-1 cells were pretreated with 30 μM IκBα inhibitor BAY11-7082 for 30 min and then stimulated with 10 μg/ml CPSIT_P7 for 24 h or 36 h. **(A,B)** RT-qPCR and ELISA were used to detect the IL-6, IL-8, and MCP-1 mRNA expression levels and IL-6, IL-8, and MCP-1 protein production. **(C)** THP-1 cells were stimulated with CPSIT_P7 (10.0 μg/ml, 6 h). Cells were stained with anti-NF-κB p65 antibody, followed by fluorescein-labeled secondary antibody incubation. Images were procured using a confocal microscope (10 × 100). Data are the means ± SD of three independent experiments. ^#^*P* < 0.05 vs. PBS group, *P < 0.05, **P < 0.01 vs. positive control group (10 μg/ml CPSIT_P7).

Likewise, pretreatment with 30 μM BAY11-7082 caused significant decreases in CPSIT_P7-induced IL-6, IL-8, and MCP-1 production (Figures 10A,B). We further investigated whether NF-κB was involved in the production of IL-6, IL-8, and MCP-1. The result showed that treatment with CPSIT_P7 resulted in a marked increase in the NF-κB immunofluorescence signal in the nucleus, indicating a strong translocation of NF-κB from the cytoplasm into the nuclear areas in CPSIT_P7-treated THP-1 cells (Figure 10C). In addition, the nuclear translocation of NF-κB was significantly abolished after the cells were pretreated with BAY11-7082 (Figure 10C).

## Discussion

Compared to the pathogenic mechanisms of other *Chlamydia*, there is little known about the pathogenesis of *C. psittaci*. Less severe, clinically ignored infections often lie underneath and are misdiagnosed due to similar symptoms caused by other respiratory causative agents (Harkinezhad et al., 2009). Hence, consciousness of the harm associated with *Chlamydia* infection and early diagnosis are significant. However, we have yet to identify the precise proteins of *C. psittaci* plasmid-encoded proteins for triggering inflammatory responses. It is known that plasmid-encoded proteins not only are a crucial virulence factor for a variety of species of *Chlamydia* but also are a major inducer of host inflammatory responses (Liu et al., 2014; Shao et al., 2017). In the present study, we sought to test the role of *C. psittaci* plasmid-encoded protein CPSIT_P7 in triggering inflammation.

It has been proved that plasmid-encoded protein protects against many pathogens (Zhou et al., 2013; Cao et al., 2015). It is not clear whether TLRs can be stimulated by CPSIT_P7 and the information on the functionality of the plasmid-encoded protein CPSIT_P7 is also very limited. A study by Zhou H et al., demonstrated that Pgp3 protein induces the production of TNF-α, IL-1β, and IL-8 via the TLR2 signaling pathway in monocytes, which could contribute to the pathogenesis of *C. trachomatis* (Zhou et al., 2013). It has been reported that TLR4 plays essential roles in the recognition of *Chlamydia* infection (Nosratababadi et al., 2017). Massari et al. (2013) revealed that the activation of immune responses via recognition of *Chlamydia* species is dependent on TLR4. Thus, it appears that TLRs may be considered as significant sensors for the identification of plasmid-encoded protein and the acceleration of inflammatory responses. In the present study, our results indicated that CPSIT_P7 stimulates monocytes for the production of these inflammatory cytokines in a TLR4-dependent manner, which is consistent with previous findings that TLRs were essential for the development of inflammation induced by chlamydial plasmid-encoded protein. To our surprise, TLR2 was not involved in CPSIT_P7-induced inflammation. We speculate that TLR2 may mainly play a role in membrane protein-induced inflammatory responses, while CPSIT_P7 is a secretory protein.

A recent finding in our work is that Mal is important for the secretion of IL-6, IL-8, and MCP-1 mediated by CPSIT_P7. Mal recruits MyD88 to the plasma membrane via TLR2 and TLR4 signaling, indicating that Mal plays an essential role in TLR signaling though the MyD88-dependent pathway. This is verified by the result that the TIR-domain surface of MyD88 is electropositive, and the surface of Mal is predicted to be electronegative (Xu et al., 2000). Furthermore, Mal is also situated upstream of MyD88 in MALP-2-induced signaling (Santos-Sierra et al., 2009). Moreover, the cytoplasmic adaptor Mal recruits the TLR2/6 heterodimer in response to MALP-2 stimulation (You et al., 2014). Our observations further support the viewpoint that Mal could downregulate the mRNA expression of MyD88 while Mal was somewhat inhibited to different degrees. To the best of our knowledge, this is the first study to verify that Mal is related to the expression of IL-6, IL-8, and MCP-1 stimulated by CPSIT_P7. Understanding the signaling pathways and cellular responses induced by CPSIT_P7 is a subject of great interest.

Growing evidence indicates that NF-κB plays an essential role in the immune and inflammatory responses by mediating cell signaling (Sellami et al., 2014). A previous study has shown that *Chlamydia* could activate NF-κB and the resulting expression of MCP-1 in human endothelial cells during the early stages of atherogenesis, which may take part in monocyte–macrophage recruitment (Rasmussen et al., 1997). In addition, p50–p65 heterodimers were identified as the active NF-κB components present in smooth muscle cells and endothelial cells infected by *C. pneumoniae* (Dechend et al., 1999), and the p65 component of NF-κB has been regarded as the principal regulator of transcriptional activation (Schmitz and Baeuerle, 1991), and the translocation of p65 from the cytoplasm to the nucleus following IκBα dissociation (Hayden and Ghosh, 2008). In the present study, we observed that NF-κB was activated by CPSIT_P7. As shown in Figure 6, nuclear translocation of p65, IκBα degradation, and phosphorylation of IκBα occur in CPSIT_P7-stimulated THP-1 cells. Furthermore, NF-κB inhibitor and BAY11-7082 suppressed IL-6, IL-8, and MCP-1 expression, indicating that NF-κB activation is required for the CPSIT_P7-induced IL-6, IL-8, and MCP-1 expression in human monocytes.

There are some limitations to this study. We used immortalized human monocyte and THP-1 cell lines; the effects of CPSIT_P7 activation on TLR4 and the subsequent release of inflammatory cytokines in humans are still unknown. In addition, it will be worth testing whether CPSIT_P7-mediated promotion of inflammation requires the tumor necrosis factor receptor 1 (TNFR1) pathway. The role of TNFR1 in chlamydial pathogenesis (Dong et al., 2014) is supported by the results that (i) the Pgp3 C-terminal trimerization domain is similar to the receptor-binding domain of TNF-α (Zhong, 2017), and (ii) Pgp3 is known to activate macrophages and dendritic cells to produce cytokines (Li et al., 2008). As mentioned above, CPSIT_P7 is homologous to the plasmid Pgp3 of *C. trachomatis*. Therefore, determining the hypothesis of CPSIT_P7 may importantly promote the understanding of chlamydial pathogenic mechanisms. At last, we only detected the role of the NF-kB signaling pathway on the inflammatory response induced by CPSIT_P7 protein, which must have other signaling pathways involved in the CPSIT_P7-mediated inflammatory response. For instance, MAPK cascade is an important signal transduction pathway, which contributes to the production of cytokines during chlamydial infection (Mark et al., 2010). Thus, CPSIT_P7 elicits inflammatory response in human monocytes, and whether it activates MAP kinase is also worth determining.

In the current study, we illustrated for the first time that the TLR4/Mal/MyD88/NF-κB signaling axis is involved in *C. psittaci* plasmid-encoded protein CPSIT_P7 stimulation in THP-1 cells. In conclusion, this study is the first report about a *C. psittaci* plasmid-encoded protein CPSIT_P7 displaying proinflammatory properties. These findings may provide a novel insight into the molecular pathogenesis of *C. psittaci* infection.

## Materials and Methods

### Reagents

The human monocytic cell line (purchased from CTCC, Wuhan University, Wuhan, China) was cultured as previously described (Xie et al., 2017). psiRNA-hTLR2, psiRNA-hTLR4, psiRNA-hTLR6, pZERO-hTLR2, pZERO-hTLR4, pZERO-hTLR6, pDeNy-hMyD88, pDeNy-hMal, and control plasmids (psiRNA-LucGL3, pZERO-mcs, and pDeNy-mcs) were obtained from InvivoGen (Carlsbad, United States). The inhibitor of NF-κB, BAY11-7082, was obtained from Cell Signaling Technology (Beverly, MA, United States). Anti-phosphorylated and anti-total IκBα and p65 mouse antibodies were obtained from Abcam (Cambridge, MA, United States). Anti-β-actin antibody was bought from Sigma-Aldrich (St, Louis, MO, United States). Cy3-conjugated goat anti-mouse IgG antibody was obtained from Invitrogen (Frederick, MD). Antibodies against TLR2, TLR4, TLR6, MyD88, and Mal were purchased from Abcam (Cambridge, MA, United States). Neutralizing antibodies against TLR2, TLR4, and TLR6 were obtained from Novus. Complete protease inhibitor cocktail was purchased from Roche Applied Science (Mannheim, Germany). Other reagents were purchased from Thermo Fisher Scientific (Waltham, United States).

### Purification of the Recombinant CPSIT_P*7*

Recombinant protein CPSIT_P7 was purified as previously described (Tan et al., 2018), and the purity of the CPSIT_P7 protein was detected by SDS-PAGE. Then, the purified CPSIT_P7 protein was treated with the ToxinEraser^TM^ Endotoxin Removal Kit (GenScript, Piscataway, United States), and the endotoxin level was measured using Limulus amebocyte lysate (Chinese Horseshoe Crab Reagent Manufactory, Ltd., Xiamen, China). The endotoxin was found to be less than 0.04 endotoxin units (EU)/ml.

### Cytokine Arrays

Cytokines secreted from human monocytic cells were determined using hybridizing medium with antibody-coated membranes (Cytokine Human Membrane Antibody Array Kit, Abcam, Cambridge, MA) according to the instructions from the manufacturer. Briefly, cells were added to serum-free medium in six-well plates and stimulated with CPSIT_P7 and cultivated overnight. The culture supernatants were collected, pooled (*n* = 3/group), and screened for inflammatory cytokines, and then mixed with the array membrane. A biotin-conjugated secondary antibody was used to detect cytokines by HRP-conjugated streptavidin. Signals were detected with an enhanced chemiluminescence system (Syngene, Cambridge, United Kingdom).

### Transient Transfection

THP-1 cells (1 × 10^6^ cells/well) were transfected with 4 μg of interfering plasmids (psiRNA-hTLR4, pZERO-hTLR2, pDeNy-hMyD88, pDeNy-hMal; psiRNA-LucGL3, pZERO-mcs, and pDeNy-mcs were used as control plasmids) using Lipofectamine 2000 (Invitrogen, Carlsbad, United States) for 24 h or 48 h. A total of 1 × 10^6^ THP-1 cells were transfected with 4 μg of pZERO-hTLR2, pZERO-hTLR4, pZERO-hTLR6, pDeNy-hMyD88, and pDeNy-hMal interfering plasmids and 4 μg of psiRNA-hTLR2, psiRNA-hTLR4, and psiRNA-hTLR6 interfering RNAi (pZERO-mcs and pDeNy-mcs were used as control plasmids; psiRNA-LucGL3 was used as control RNAi). After 24 or 48 h of transfection, cells were stimulated for 24 or 36 h with CPSIT_P7 (10 μg/ml).

### Western Blot

Cells were washed with phosphate-buffered saline (PBS) and harvested in lysis buffer (containing protease and phosphatase inhibitor) (Beyotime Institute of Biotechnology, Jiangsu, China). After centrifugation at 4^*o*^C at 12,000 × *g* for 15 min, samples were transferred to nitrocellulose membranes after denaturation and subjected to SDS-PAGE. The membranes were blocked with 5% non-fat milk followed by incubation with specific indicated antibodies overnight. After incubation with secondary antibodies for 1 h, the following antibodies were used: TLR2 antibody (1:1000 dilution), TLR6 antibody (1:500 dilution), MyD88 antibody (1:1500 dilution), Mal (1:1500 dilution), IκBα antibody (1:1500 dilution), and p-IκBα antibody (1:1500 dilution). β-actin (1:1000 dilution) was used as internal control protein. Protein bands were determined with an enhanced chemiluminescence system and were analyzed by ImageJ software.

### RT-qPCR

Total RNA was isolated using TRIzol Reagent (Invitrogen, Carlsbad, CA) from THP-1 cells stimulated with either CPSIT_P7 or *Escherichia coli* LPS. cDNA was synthesized according to the manufacturer’s instructions. The primer sequences used for amplifications were as follows (Xu et al., 2017): IL-6 forward, 5′-TACATCCTCGACGGCATCTC-3′; IL-6 reverse, 5′-TTTCAGCCATCTTTGGAAGG-3′; IL-8 forward, 5′-AGCTCTGTGTGAAGGTGCAGT-3′; IL-8 reverse, 5′-AATTTCTGTGTTGGCGCAGT-3′; MCP-1 forward, 5′-AATCCAGCTCCTTCCAGGAT-3′; MCP-1 reverse, 5′-ACACACCCACCCTCTCTTTG-3′; TLR2 forward, 5′-ACTTCATTCCTGGCAAGTGG-3′; TLR2 reverse, 5′-TTTTTCTCAATGGGCTCCAG-3′; TLR4 forward, 5′-CCTGTCCCTGAACCCTATGA-3′; TLR4 reverse, 5′-TCTAAACCAGCCAGACCTTGA-3′; TLR6 forward, 5′-TGAATGCAAAAAAACCCTTCAC-3′; TLR6 reverse, 5′-CCAAGTCGTTTCTATGTGGTT-3′; MyD88 forward, 5′-CCGCCTGTCTCTGTTCTTG-3′; MyD88 reverse, 5′-GTCGCTTGTGTCTCCAGTT-3′; GAPDH forward, 5′-CAGGAGGCATTGCTGATGAT-3′; GAPDH reverse, 5′-GAAGGCTGGGGCTCATTT-3′. Real-time quantitative PCR (RT-qPCR) was performed using a LightCycler 96 apparatus (Roche, Basel, Switzerland) and SYBR green (Qiagen, Shanghai, China). The specificity of the PCR was controlled by no-template controls. Each RNA sample was tested in triplicate. Data were analyzed by the 2^–△△*Ct*^ method and normalized to the housekeeping gene GAPDH.

### Enzyme-Linked Immunosorbent Assay

The levels of IL-6, IL-8, and MCP-1 in supernatants were determined by ELISA Kits (Affymetrix, eBioscience, Santa Clara, CA, United States). The assay sensitivities were 2 pg/ml (IL-6), 2 pg/ml (IL-8), and 7 pg/ml (MCP-1), respectively. All experiments were repeated three times.

### NF-κB Immunofluorescence

Cells were seeded in 35-mm dishes and then fixed with 4% paraformaldehyde at 4°C for 30 min. Cells were permeabilized with 0.5% Triton X-100 for 15 min at room temperature. After blocking with 10% fetal bovine serum in RPMI 1640 medium for 1 h, the cells were incubated with a polyclonal antibody against p65 (1:200 dilution) and then incubated with a secondary Cy3-conjugated goat-anti-rabbit antibody diluted at 1: 200 in medium for 1 h at room temperature. The cells were then washed and stained with DAPI (1 mg/ml) for 1 h. The result was observed under an immunofluorescence microscope (Nikon, Japan).

### Statistical Analysis

The results of all experiments are reported as the mean values ± SEM of at least three independent experiments. GraphPad Prism 6.0 software (GraphPad Software, Inc., La Jolla, CA) was used for comparison between two groups. *P* < 0.05 was considered as statistically significant.

## Data Availability Statement

The data used to support the findings of this study are included within the article and the Supplementary Material.

## Author Contributions

QC designed the experiments. YL drafted the manuscript. XY and ZS performed experiments and analyzed cell experimental data. CW, SL, and JX analyzed experimental results and data. CL and YW guided the design of the study and revised the manuscript. All authors read and approved the final manuscript, contributed to the article and approved the submitted version.

## Conflict of Interest

The authors declare that the research was conducted in the absence of any commercial or financial relationships that could be construed as a potential conflict of interest.
