# Supplementary figures and images for: Chlamydia psittaci Plasmid-Encoded CPSIT_P7 Elicits Inflammatory Response in Human Monocytes via TLR4/Mal/MyD88/NF-κB Signaling Pathway
Source: Front Microbiol. 2020 Dec 3;11:578009. doi: 10.3389/fmicb.2020.578009 (PMC7744487; doi:10.3389/fmicb.2020.578009)

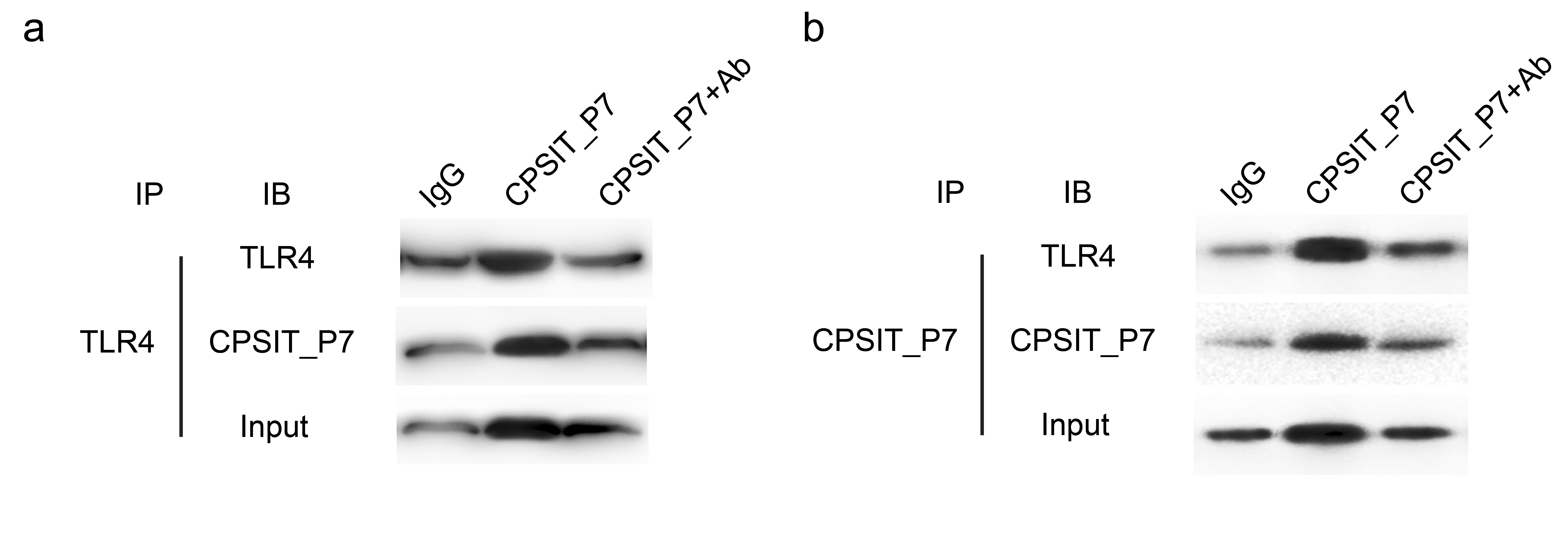

Supplement: Supplementary file 1 [file Image_1.TIF]
